# Effect and Mechanism of Qihua Tongtiao Formula (QHTTF) on Improving Glucose and Lipid Metabolism Disorders in ZDF Rats by Integrating Network Pharmacology, Metabolomics, and Biological Validation

**DOI:** 10.3390/ph18091347

**Published:** 2025-09-08

**Authors:** Yuhua Jiang, Hong Yu, Yajing Pan, Binghan Zhang, Yeteng Jing, Jingjing Lei, Ning Li, Jinsheng Yang

**Affiliations:** 1 Institute of Basic Theory of Traditional Chinese Medicine, China Academic of Chinese Medical Sciences, Beijing 100193, China; 2 Key Laboratory of Chinese Internal Medicine, Ministry of Education and Beijing, Dongzhimen Hospital, Beijing University of Chinese Medicine, Beijing 100700, China; 3 Department of Pharmacology, University of Texas Health Science Center at San Antonio, San Antonio, TX 78229, USA

**Keywords:** Qihua Tongtiao Formula, T2DM, glucolipid metabolism disorders, PPARα, PPARγ, insulin resistance

## Abstract

**Background:** The dysregulation of both glucose and lipid metabolism is the main clinical features of type 2 diabetes. Qihua Tongtiao Formula (QHTTF) is our team’s current clinical empirical formula, and the related patent has been granted. It is composed of *Astragalus membranaceus*, *Atractylodes macrocephala koidz*, *Aurantii Fructus Immaturus*, *Radix Bupleuri*, *Ligusticum chuanxiong hort*, *Angelicae sinensis radix*, *Raphanus sativus*, and *Polyporus umbellatus*. It can alleviate tissue pathological damage in type 2 diabetic rats by improving glycolipid metabolism disorders. Nevertheless, the specific mechanisms of QHTTF in the treatment of type 2 diabetes remain unclear. Purpose: This research aims to explore the fundamental effect and underlying mechanism of the QHTTF formula in ZDF rats via network pharmacology, biological validation, and metabolomics technology. **Methods:** The chemical compounds of QHTTF were initially identified via UHPLC-MS/MS analysis. Meanwhile, drug targets, genes, related diseases, and differential metabolites of QHTTF in the treatment of T2DM were obtained through network pharmacology, molecular docking, and metabolomics. Then, we conducted animal experiments to further explore the therapeutic molecular mechanism of QHTTF in ZDF rats. **Results:** A total of 39 main chemical components were recognized through LC-MS/MS technology, and 22 remained after the second screening. Network pharmacology and molecular docking results revealed that 59 intersection targets were involved in the treatment of glycolipid metabolic disorders, and the PPARα, PPARγ, and TNF proteins were identified as crucial targets through PPI network analysis. Additionally, serum metabolomics analysis of ZDF rats showed that QHTTF could regulate linoleic acid metabolism, fructose and mannose metabolism, galactose metabolism, fatty acid biosynthesis, and other related signaling pathways. The results of biological experiments proved that QHTTF effectively lowered blood glucose and lipid levels, alleviated hepatic and pancreatic pathological damage, increased the expression of IRS-1 and GLUT4 in the pancreas, and improved insulin resistance, while inhibiting the inflammatory response and oxidative stress, as well as enhancing the expression of liver PPARα, PPARγ, and AMPK proteins in ZDF rats. **Conclusions:** In summary, QHTTF exerted a significant effect in improving glycolipid metabolism disorders of ZDF rats, which might show therapeutic effects by relieving insulin resistance, mitigating inflammation and oxidative damage, regulating related glucose, fatty acid, and amino acid metabolism, and increasing the expression of PPARα, PPARγ, and AMPK proteins by combining network analysis, metabolomics, and biological research.

## 1. Introduction

Type 2 diabetes mellitus (T2DM) is a prevalent endocrine disease characterized primarily by hyperglycemia, impaired glucose tolerance, insulin resistance, and disrupted glucose and lipid homeostasis [1]. Global diabetes prevalence in 2021 was estimated to be approximately 10.5% of the population (536.6 million) and is projected to rise to 12.2% (783.2 million) by 2045, according to recent IDF statistics [2]. T2DM accounts for nearly 90% of all diabetes mellitus cases globally [3] and can lead to dysfunction of multiple organs and systems, including cardiovascular disease and nephropathy, among others. Meanwhile, an increasing severity of metabolic syndrome symptoms, such as hyperglycemia and hyperlipidemia, has become exceedingly prevalent worldwide owing to cases of overweight and obesity, with changes in dietary habits and lifestyles [4]. However, the mechanisms underlying glucose and lipid metabolism dysregulation in T2DM are complex. Previous research has indicated significant associations with systemic inflammation responses, oxidative damage, and impaired insulin sensitivity [5,6]. In addition, IRS-1 (insulin receptor substrate-1), peroxisome proliferator-activated receptors (PPARs), AMP-activated protein kinase, and other metabolic products, including lipids, amino acids, and carbohydrate metabolites, are strongly implicated in glucose and lipid metabolism disorders of diabetes mellitus [7,8]. Therefore, the prevention and management of T2DM are critically significant. During the past few decades, dietary management and medication for blood glucose and lipid reduction have been the main therapies for T2DM, including biguanides, sulfonylureas, thiazolidinediones, insulin, statins, and others. Nevertheless, most of them have failed to delay diabetes progression and exhibit adverse reactions or toxicity despite their temporary efficacy [9]. Consequently, the exploration of safe and effective glycolipid-regulating drugs with multiple target actions in the treatment of T2DM is an essential objective.

Traditional Chinese medicine (TCM) has a long history in promoting human health and treating chronic diseases based on a holistic view. It is commonly used in the form of herbal formulas, which leverage the synergistic effects among various herbs, and TCM is also known for its lower rates of drug resistance and side effects [10]. Some TCM herbs have proven to be remarkably efficient in managing T2DM and related cardiovascular illnesses, based on the pathogenesis of Qi blockage, phlegm dampness, and blood stasis accumulation in Chinese medicine [11]. Accordingly, TCM herbs can relieve symptoms and slow the progression of diabetes mellitus through therapeutic strategies such as replenishing Qi and strengthening the spleen, resolving phlegm and dampness, and dispelling stasis and eliminating turbidity. Qihua Tongtiao Formula (QHTTF) is a further modification based on Huangqi Baizhu Decoction from *the Yellow Emperor’s Inner Canon* and Zhizhu Decoction from *the Synopsis of the Golden Chamber*, while incorporating clinical treatment experience. The formula is composed of Huangqi (*Astragalus membranaceus*), Baizhu (*Atractylodes macrocephala koidz*), Zhishi (*Aurantii fructus immaturus*), Chaihu (*Radix bupleuri*), Chuanxiong (*Ligusticum chuanxiong hort*), Danggui (*Angelicae sinensis radix*), Laifuzi (*Raphanus sativus*), and Zhuling *(Polyporus umbellatus)*. It was prepared as a decoction for experimental research. In our preliminary study, QHTTF showed a significant therapeutic effect on glycemic control and lipid regulation in Sprague Dawley diabetic rats and has been proven effective in lowering fasting blood glucose and lipid levels of those with type 2 diabetes. Thus, we further conducted the experimental study presented in this paper to clarify the underlying mechanisms of QHTTF in treating glucose and lipid metabolic disorders of T2DM.

With the advent of large-scale data and artificial intelligence, the integration of informatics technologies—such as network pharmacology, molecular biology techniques, multi-omics technologies, and biological experiment methods—offers a promising approach for the comprehensive illustration of TCM formula principles [12]. In recent years, numerous studies have been conducted by combining network pharmacology methods and metabolomics, and these studies have contributed to clarifying the pathophysiology of glycolipid metabolic disorders in T2DM [13]. Notably, metabolomics examines the human body as an integrated system characterized by its holistic, dynamic, and non-invasive nature. This approach is closely aligned with the principles of holistic and dynamic concepts in TCM. Furthermore, metabolomics is generally used to describe abnormal metabolism during the progression of glycolipid metabolic disorders. Meanwhile, network pharmacology constructs interaction networks in the context of biological systems and explores the connections between multiple drug targets via the “disease-targets-drug” network [14,15]. Thus, traditional Chinese medicine and modern approaches like network pharmacology and metabolomics, with their integrated concepts, can provide insights into disease mechanisms and explore disease-related targets for T2DM treatment.

**Figure 1:**
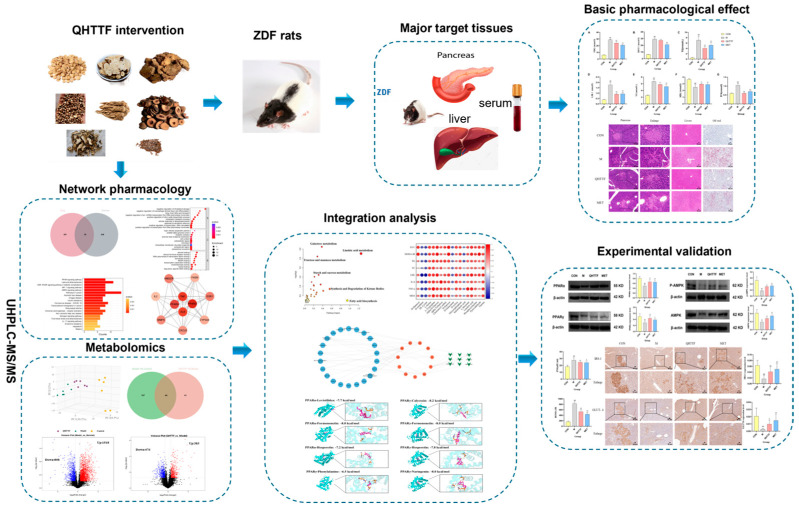
Flow diagram of the current research.

In the present study, the components of QHTTF were determined by means of ultra-performance liquid chromatography–mass spectrometry (UPLC-MS/MS), which is a high-throughput technique. Subsequently, a “compound-target-metabolite” network was established using network pharmacology combined with metabolomics to identify the primary targets and therapeutic metabolic pathways. Furthermore, an experimental assay was developed in ZDF rats induced by high-fat diets to illustrate the related mechanisms of improving glycolipid metabolic disorders. The workflow of this study is illustrated in Figure 1.

## 2. Results

### 2.1. The Main Chemical Constituents of QHTTF

**Figure 2:**
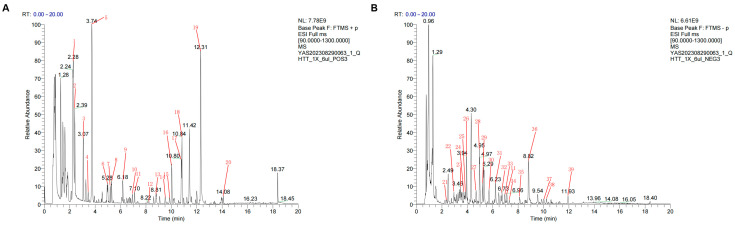
Total ion chromatogram of QHTTF in positive (**A**) and negative modes (**B**).

The main chemical compounds were identified in QHTTF under positive and negative ion scanning mode using a UHPLC-MS/MS system and were divided into seven primary categories: alkaloids, terpenoids, carbohydrates, fatty acids, polyketides, amino acids and peptides, Shikimates and Phenylpropanoids, and others. The base peak chromatogram (BPC) of QHTTF in both pos and neg modes is shown in Figure 2A,B. A sum of 39 chromatographic peaks were labeled, and 22 were further screened through Lipinski’s Rule of Five and GI absorption (Table 1). Additionally, the 39 pieces of specific information about the identified compounds are provided in Supplementary Table S1. These compounds mainly contained tryptophan alkaloids, isoflavonoids, phenylpropanoids (C6-C3), flavonoids, cyclic polyketides, small peptides, etc., in the label peaks in numerical order.

### 2.2. QHTTF Improved Glucose and Lipid Metabolism Levels in ZDF Rat Models

**Figure 3:**
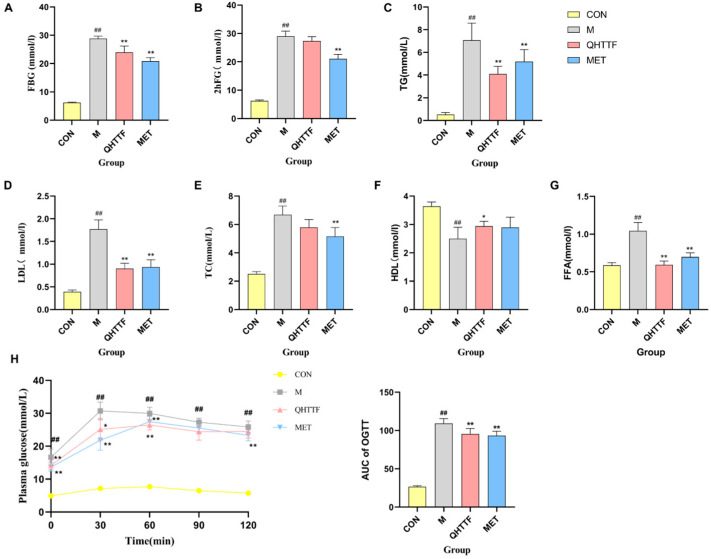
Effect of QHTTF on glucose and lipid metabolism levels: (**A**) fasting blood glucose, (**B**) 2 h postprandial blood glucose, (**C**) triglyceride, (**D**) low-density lipoprotein cholesterol, (**E**) total cholesterol, (**F**) high-density lipoprotein, (**G**) free fatty acid level, and (**H**) OGTT and corresponding AUC values ($\bar{x}$ ± s, *n* = 8). ## *p* < 0.01 vs. Con; * *p* < 0.05 and ** *p* < 0.01 vs. Model. Note: CON: Normal control ZDF rats (fa/+); Model: type 2 diabetic model; QHTTF: Traditional Chinese medicine treatment group; MET: Metformin-treated group (positive control).

After eight weeks of drug treatment, the FBG, 2hFG, TG, TC, FFA, and LDL levels were substantially higher in model rats than in the control group, while significantly decreasing in the metformin groups compared to untreated models (*p* < 0.01). Meanwhile, the levels of FBG (Figure 3A), TG (Figure 3C), LDL (Figure 3D), and FFA (Figure 3G) were substantially reduced in the QHTTF group (*p* < 0.01) compared to the models, but 2hFG (Figure 3B) and TC (Figure 3E) levels showed no significant change (*p* > 0.05). Moreover, compared with the healthy control rats, the serum HDL levels in the model group significantly decreased (*p* < 0.01), and the QHTTF group exhibited an upward trend (*p* < 0.05), whereas no statistically significant difference was observed in the metformin group (*p* > 0.05) relative to the model controls (Figure 3F).

The oral glucose tolerance testing (OGTT) results revealed differential blood glucose levels in all groups of ZDF rats. Blood glucose at various time points in the model rats was notably higher (*p* < 0.01), and the area under the curve (AUC) was markedly larger than that in the control group (*p* < 0.01). After drug treatment, the AUC of the OGTT in the QHTTF group and the metformin group was significantly reduced compared with the model group (*p* < 0.01), and blood glucose levels peaked at 60 min after administration. Moreover, blood glucose levels showed a downward trend at 90 and 120 min, but there was no statistically meaningful difference in QHTTF (*p* > 0.05) (Figure 3H).

### 2.3. QHTTF Regulated Body Weight and Liver Weight Levels in ZDF Rat Models

**Figure 4:**
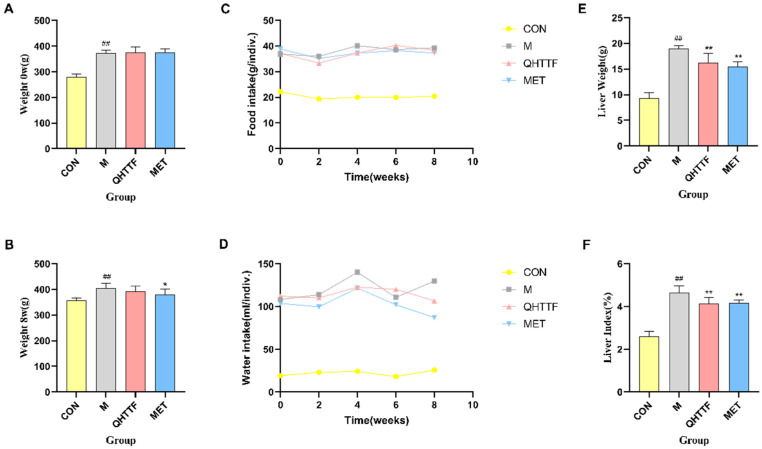
Effect of QHTTF on the body mass, dietary and water intake, and liver weight of ZDF rats. (**A**,**B**) body weight (0, 8 w); (**C**) trend of changes in food intake; (**D**) trend of changes in water intake (*n* = 8); (**E**,**F**) liver weight and liver index. ## *p* < 0.01 vs. Con; * *p* < 0.05 and ** *p* < 0.01 vs. Model.

As shown in Figure 4A,B, the body mass of the healthy control was markedly reduced versus the model rats (*p* < 0.01) before the drug intervention, while QHTTF and Met-treated groups showed comparable baseline weights to the models, indicating that the treatments had no significant impact on body weight between the groups (*p* > 0.05) (Figure 4A). Following 8-week treatment, model rats exhibited significantly higher body weight than the controls (*p* < 0.01). The body weight in the metformin group was significantly lower than that of the models (*p* < 0.01), while there was no significant difference in the QHTTF group (*p* > 0.05) (Figure 4B). Meanwhile, relative to the controls, model group rats exhibited significantly elevated food and water consumption. However, the change trend in food intake were not apparent relative to the model after QHTTF and metformin interventions, while a decreasing trend in water consumption was observed, particularly at the 4th and 8th weeks (Figure 4C,D). Notably, the data represent the average food/water intake per cage (not per rat); thus, statistical comparisons are not applicable. Additionally, the liver weight (Figure 4E) and liver index (Figure 4F) of the model group rats were significantly increased (*p* < 0.01) relative to the control group, and the QHTTF and Met group showed a significant reduction in liver weight and liver index compared with the model group (*p* < 0.01), indicating that the traditional Chinese medicine formula can reduce the liver weight and liver index of ZDF model rats (Figure 4E,F).

### 2.4. QHTTF Alleviated Pathological Damage to the Liver and Pancreas Tissues

**Figure 5:**
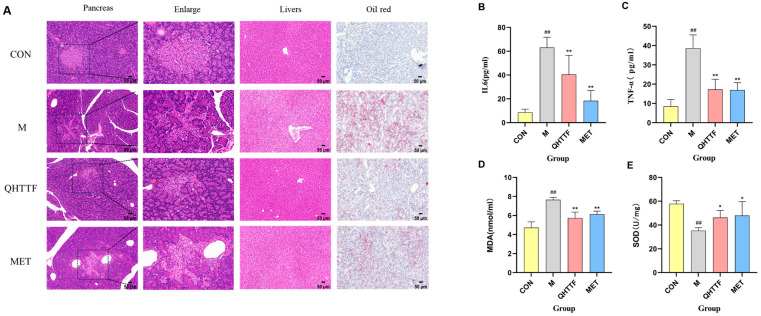
Effect of QHTTF on pathological damage in ZDF rats: (**A**) HE staining of the pancreas and livers, with Oil Red O for the livers (200×, *n* = 6, 50 μm); (**B**,**C**) serum inflammatory factors IL-6 and TNF-α levels; (**D**,**E**) oxidative stress indicators MDA and SOD levels. ($\bar{x}$ ± s, *n* = 6), ## *p* < 0.01 vs. Control; * *p* < 0.05, ** *p* < 0.01 vs. Model.

As shown in Figure 5A, pancreatic HE staining results indicated that in the healthy group, the pancreatic morphology was intact, with regular arrangement of islet cells, clear boundaries, and uniform distribution. The model group showed irregular pancreatic morphology, disorganized structure, unclear boundaries, and islet cells of uneven size, with some cells undergoing degeneration or necrosis compared to the healthy rats, and the islet morphology in the QHTTF group and the Met group showed significant improvement, with more regular arrangement, and a notable reduction in cell vacuolation and necrosis. Furthermore, liver H&E staining revealed that model rats displayed abnormal cellular morphology, unclear liver sinusoids, with a large amount of fat vacuolation, degeneration, and inflammatory infiltration. In contrast, in the QHTTF group, the arrangement of liver cell cords was more orderly, with a significant reduction in inflammatory infiltration and cell vacuolation degeneration, and clear liver sinusoids were observed. Meanwhile, liver Oil Red O staining results revealed that there were numerous bright red fat droplets visible within hepatocyte cytoplasm, diffusely distributed in the model group, indicating severe lipid accumulation and the presence of fatty degeneration. In the QHTTF group, the number of fat droplets in liver cells was significantly reduced, with some dark red fat droplets visible in the cytoplasm, indicating a reduction in lipid accumulation and a significant improvement in fatty degeneration.

### 2.5. QHTTF Suppressed Inflammation Reactions and Oxidative Damage in ZDF Rats

The effects of QHTTF on inflammation and oxidative stress are presented in Figure 5B–E. Compared to the healthy control group, the serum levels of interleukin-6 (IL-6) (Figure 5B), tumor necrosis factor-α (TNF-α) (Figure 5C), and the lipid peroxidation marker malondialdehyde (MDA) (Figure 5D) were significantly elevated in the model group (*p* < 0.01), while the superoxide dismutase (SOD) (Figure 5E) level was significantly lower than in healthy rats. After intervention with the QHTTF, the levels of IL-6, TNF-α, and MDA were significantly reduced in the model group, while the SOD level was significantly higher than that of the models (*p* < 0.01). These results suggest that QHTTF can reduce the levels of inflammatory mediators and oxidative stress, thereby improving the dysregulation of glucolipid metabolism and tissue damage in ZDF rats.

### 2.6. Network Construction and Key Target Discovery of QHTTF

**Figure 6:**
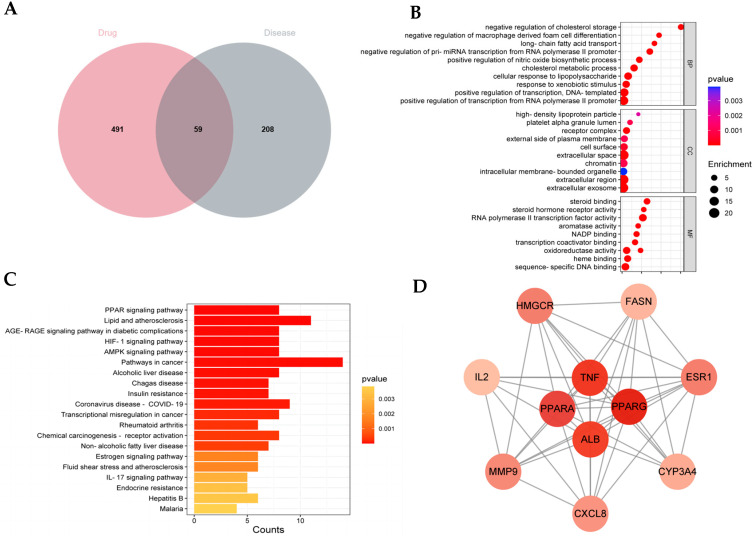
Network pharmacology analysis of QHTTF: (**A**) Venn diagram between the QHTTF and T2DM with hyperlipidemia targets. (**B**) The top 10 BPs, MFs, and CCs from the GO analysis. (**C**) The top 20 pathways from the KEGG analysis. (**D**) PPI network of core targets for JSHF in the treatment of TZDM with hyperlipidemia.

The 22 main compounds left were retrieved and searched in Herb, PubChem, and Swiss ADME databases with five drug-screening rules. The targets were predicted through the Swiss Target Prediction database, and the screening criterion was a probability > 0.1, thereafter standardized by using the UniProt database. Ultimately, 550 targets were obtained in total after removing duplicates. On the other hand, the disease targets for T2DM with hyperlipidemia were searched from the Drug Bank, OMIM, and Gene Card databases. After standardization, 267 potential targets were ultimately screened. Therefore, to evaluate the anti-glucolipid action of QHTTF, a total of 59 common targets between QHTTF and T2DM with hyperlipidemia were identified and visualized by using a Venn diagram, as shown in Figure 6A.

Next, the 59 intersection targets were analyzed using the STRING data platform (https://cn.string-db.org) to built a protein interaction network of QHTTF and glycolipid metabolism diseases, which included 59 nodes and 494 edges, with an average node degree of 16.7 and an average clustering coefficient of 0.615 (all *p*-values were less than 0.05). Subsequently, a TSV file was loaded and imported into Cytoscape 3.9.1 software with the CentiScape plug-in, which filtered based on the three values: Degree (>17.03448275862069), Closeness (>0.010220051786122106), and Betweenness (>42.517241379310335). Finally, the PPI core network was constructed, and, ultimately, 11 core target proteins (like PPARα, PPARγ, TNF, ALB, and so on) were identified through statistical analysis (Figure 6D).

In addition, GO and KEGG pathway enrichment analyses were performed to gain further insights into the common targets and genes from the David database. As indicated by the data, there were 191 biological processes (BPs), 60 molecular functions (MFs), and 15 cellular components (CCs) in GO analysis (*p* < 0.05), which mainly contained cholesterol metabolic processes, cellular response to lipopolysaccharide, response to xenobiotic stimulus, steroid binding, oxidoreductase activity, and other biological functions. The top 10 results for BPs, MFs, and CCs are shown with a bubble graph in Figure 6B. Simultaneously, 68 total items were identified in KEGG analysis. The primary 20 pathways were screened out based on *p*-value (*p* < 0.05) and predominantly included the PPAR signaling pathway, insulin resistance, lipid and atherosclerosis, the AMPK pathway, alcoholic liver disease, and the IL-17 pathway, among others. The 10 top-ranking pathways are visualized and depicted with the KEGG bar diagram (Figure 6C).

### 2.7. Results of Molecular Docking of QHTTF

**Figure 7:**
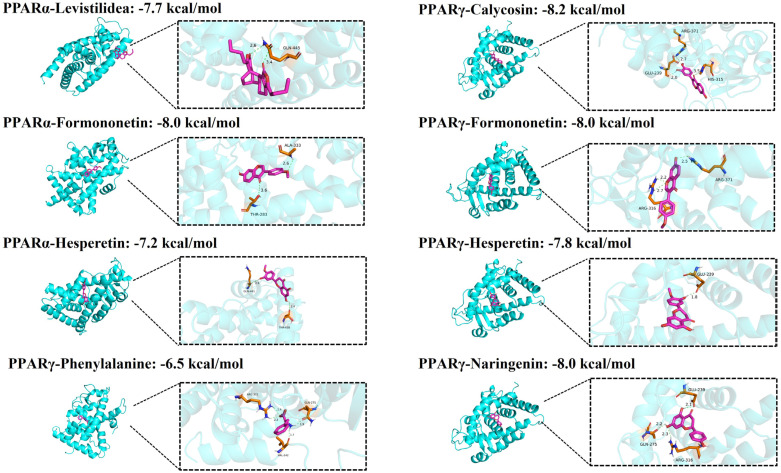
Molecular docking analysis of 8 combinations with high binding energy. (Calycosin, Formononetin, Phenylalanine, Naringenin, Hesperetin and Levistilide A bind to PPARα and PPARγ).

The nine most significant active components in QHTTF were selected for molecular docking with the core targets PPARα, PPARγ, and TNF. Our results demonstrated strong interactions and stable binding between the ligands and proteins, with all binding energies below −4 kcal/mol. Notably, calycosin, formononetin, phenylalanine, naringenin, hesperetin, and Levistilide A exhibited binding energies below −6 kcal/mol for both PPARα and PPARγ, indicating high binding activity between QHTTF components and core targets (Figure 7).

### 2.8. Metabolic Serum Profiling of QHTTF

**Figure 8:**
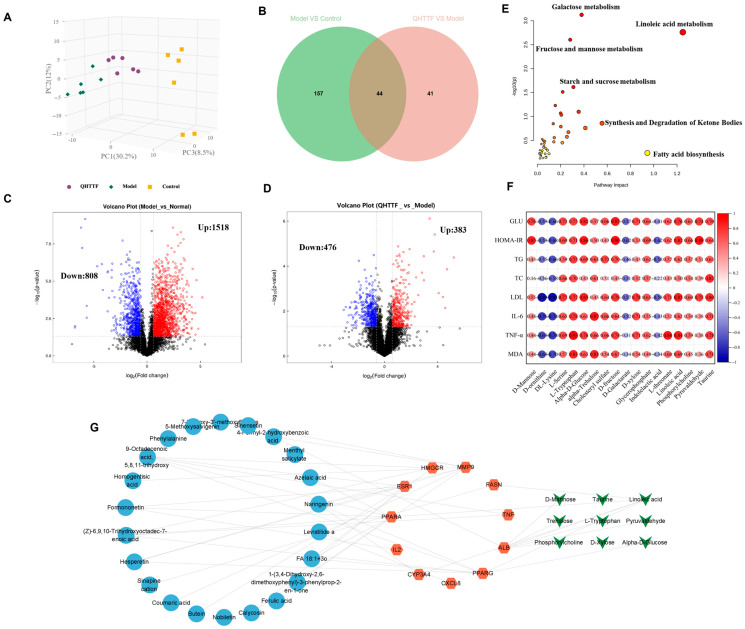
Metabolome profiling for QHTTF in ZDF rats. (**A**) PCA score plots. (**B**) A Venn diagram of the serum metabolites among groups. (**C**,**D**) Volcano plots. (**E**) The major metabolism pathways in rat serum. (**F**) The heatmap analysis of serum metabolic markers and biochemical indicators. (**G**) The networks of key components–targets–metabolites.

A total of 1177 metabolites were identified under positive and negative ion modes according to the results of serum untargeted metabolomics. Principal component analysis was utilized to assess the distribution trend of samples and the degree of differentiation between groups. As shown in Figure 8A, three groups were clearly separated between the Con, M, and QHTTF groups (R^2^X = 0.507), which indicated dysregulation caused in the metabolic profile of ZDF rats on account of glucose–lipid metabolism disorder. Additionally, volcano plots showed the upregulation (in red) and downregulation (in blue) of metabolites across the groups (Figure 8C,D), which revealed that QHTTF has a regulating effect on the metabolomic changes in ZDF rats. Meanwhile, OPLS-DA, Orthogonal Partial Least Squares Discriminant Analysis, was employed to examine alterations in serum metabolites. There were 201 differential metabolites determined between the model and control groups, while 85 differential metabolites were identified in the model and QHTTF groups through OPLS-DA models (VIP > 1.0, *p* value < 0.05). Accordingly, 44 different metabolites in the serum between three groups were defined and displayed using Venn analysis (Figure 8B), and the specific information of differential metabolites was recorded in Supplementary Table S2.

Subsequently, the 44 common differential metabolites and the 59 intersecting target genes related to drugs and diseases were imported into MetaboAnalyst 6.0 for integrated analysis. As shown in the results, the metabolic pathway primarily included linoleic acid metabolism, fructose and mannose metabolism, galactose metabolism, fatty acid biosynthesis, synthesis and degradation of ketone bodies, and starch and sucrose metabolism, which were closely associated with the glucose and lipid metabolism of rats (Figure 8E). At the same time, the heatmap in Figure 8F shows the relationships between the biochemical markers and part major differential metabolites in the model and treatment groups to further investigate the effects of QHTTF. The Spearman’s correlation analysis in the serum of ZDF rats presented a substantial positive correlation between GLU, HOMA-IR, TC, TG, and LDL-C with the relative abundance of D-fructose, alpha-D-Glucose, L-serine, L-tryptophan, Linoleic acid, Phosphorylcholine, and Pyruvaldehyde, while exhibiting a notable negative link with the metabolites of D-ornithine, DL-Lysine, D-Galactarate, and Indolelactic acid. Furthermore, there were also significant positive correlations between inflammatory and oxidative stress markers, including IL-6, TNF-α, MDA with L-tryptophan, α-D-Glucose, α-Trehalose, D-fructose, Linoleic acid, and Taurine, and conversely, strong negative correlations with D-ornithine and DL-Lysine.

Lastly, an interaction network of “compound-target-metabolite” of QHTTF was constructed using Cytoscape 3.9.1 software to elucidate the mechanism of QHTTF for glucose and lipid metabolism diseases. As the results show, the chemical compounds, primarily including calycosin, formononetin, levistilide A, hesperetin, naringenin, and azelaic acid, in the formula might regulate Linoleic acid, Phosphorylcholine, α-D-Glucose, and D-Mannose by influencing related targets involved in PPARA (α), PPARG (γ), ALB, and others and finally trigger glucose and lipid metabolism changes (Figure 8G).

### 2.9. QHTTF Regulated Insulin Resistance and Signaling Pathway-Related Targets in Rats

**Figure 9:**
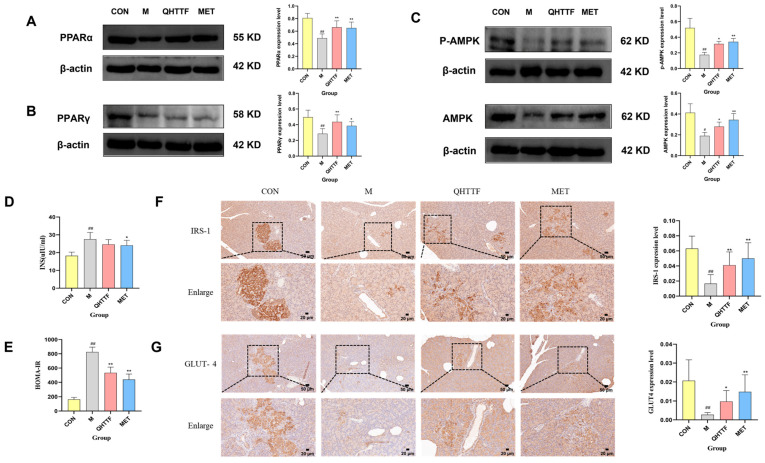
Effect of QHTTF on insulin resistance and PPAR and AMPK protein expression in rats. (**A**,**B**) The expression of PPARα and PPARγ proteins; (**C**) P-AMPK and AMPK protein expression (*n* = 6); (**D**,**E**) serum insulin and IR index levels; (**F**,**G**) the expression of IRS-1 and GLUT4 proteins (200× *n* = 6, 50 μm). # *p* < 0.05 and ## *p* < 0.01 vs. Con; * *p* < 0.05 and ** *p* < 0.01 vs. Model.

The serum biochemical analysis showed that the fasting insulin level (FINS) (Figure 9D) in model rats significantly increased (*p* < 0.01), while it decreased in the QHTTF group, though not in a statistically significant manner (*p* > 0.05). However, compared with model rats, the HOMA-IR (Figure 9E) level in both the QHTTF and Met groups significantly decreased (*p* < 0.01). Thus, QHTTF could lower the insulin resistance index and improve insulin resistance in ZDF rats. Additionally, the immunohistochemical staining results of IRS-1 (Figure 9F) and GLUT4 (Figure 9G) proteins in pancreatic tissue are displayed in Figure 9F,G. In the healthy control group, the expression levels of IRS-1 and GLUT4 proteins in the pancreas were relatively high, while the expression levels in the models were markedly lower than in the healthy controls (*p* < 0.01). After treatment with QHTTF, the expression levels of IRS-1 were significantly higher compared to the model group (*p* < 0.01), and the expression of GLUT4 exhibited the same trend (*p* < 0.05). The results indicated that QHTTF could enhance the expression of IRS-1 and GLUT4 proteins in the pancreas to some extent, which may be associated with an improvement in insulin resistance.

### 2.10. QHTTF Upregulated the Expressions of PPARα and PPARγ Proteins in the Liver

To further explore the underlying mechanism of the traditional Chinese medicine formula QHTTF, Western blotting was performed to measure the expression of peroxisome proliferator-activated receptors PPARα (Figure 9A), PPARγ (Figure 9B), and AMPK, P-AMPK (Figure 9C) proteins in liver tissue. The results are shown in Figure 9A–C, which indicates that the expression of PPARα, PPARγ, and AMPK in model rats was markedly lower than in the controls (*p* < 0.01). However, the QHTTF group showed a certain degree of increase in the expression of PPARα and PPARγ, as well as AMPK and P-AMPK proteins (*p* < 0.05), suggesting that the traditional Chinese medicine formula may improve glucose and lipid metabolism disorders in ZDF rats by regulating the expression of hepatic PPARα, PPARγ, and AMPK proteins.

## 3. Discussion

Glycolipid metabolic disorders (GLMDs) represent metabolic dysfunctions caused by impaired glycolipid homeostasis. Diabetes independently contributes to ASCVD risk, and dyslipidemia plays a crucial role in the occurrence of ASCVD in diabetic populations. Diabetic individuals, particularly T2DM patients, exhibit markedly higher dyslipidemia rates than non-diabetic controls, constituting a major risk in ASCVD and diabetes progression [16]. Diabetic dyslipidemia is caused by multiple complex pathophysiological mechanisms such as insulin resistance, chronic inflammation, oxidative stress, and energy metabolism disorders [7,17]. At present, an individual intervention is insufficient to manage multiple metabolic disorders effectively, leading to inadequate lipid management and blood sugar regulation. Based on extensive clinical experience, TCM has developed numerous effective prescription drugs under the guidance of a holistic view and dialectical treatment principles. These formulas inherit ancient formulations, are applied in modern diseases, preserve their essence, and further innovate. From the perspective of TCM theory, T2DM patients with dyslipidemia commonly have Qi deficiency in the spleen and stagnation in the liver. Deficiency and obstruction of Qi often lead to blood stagnation, damp accumulation, and toxic obstruction. Therefore, a herbal compound—QHTTF was developed based on the pathogenesis of the disease and in conjunction with classical Chinese herbal formulas, which exerts effects of strengthening the spleen and resolving dampness, smoothing the liver and regulating Qi, and eliminating turbidity in GLMDs.

The Zucker Diabetic Fatty rats model, induced by high-fat feeding, is commonly used for research on diabetes and obesity. This model is characterized by a genetic mutation in the leptin receptor, leading to hyperglycemia, obesity, hyperinsulinemia, hyperlipidemia, and impaired glucose tolerance [18,19]. It is widely employed in research on metabolic syndrome, including diabetes and obesity-related diseases. In our research, we selected Qihua Tongtiao Decoction and Metformin for intervention in ZDF rats to evaluate the effects of Chinese herbal compounds on glucose and lipid metabolism disorders, as well as the underlying mechanisms. Meanwhile, network pharmacology further identified the active ingredients, major targets, and signaling pathways of QHTTF related to the disease, while exploring mechanisms of disease progression and drug action. Broadly targeted metabolomics was also used to assess metabolite differences under various physiological and disease states. Accordingly, by combining the two methods and integrating biological experiments, we further revealed the mechanisms of QHTTF.

In the present study, we identified 39 active components of QHTTF using UHPLC-Q-MS and further screened 22 major active compounds based on pharmaceutical principles, including Lipinski’s Rule of Five and GI absorption. In this way, calycosin, phenylalanine, hesperetin, naringenin, levistilide A, ferulic acid, sinapine cation, and coumaric acid were identified as the primary active ingredients of QHTTF. These components are derived from Chinese herbs such as *Astragalus*, *Atractylodes*, *Citrus aurantium*, *Ligusticum chuanxiong*, *Angelica sinensis*, *Raphanus sativus*, *Bupleurum*, and others based on the 2020 edition of the *China Pharmacopoeia*. In particular, most of these components have been reported to influence glucose and lipid levels and ameliorate issue and organ damage in rats with T2DM or dyslipidemia. For instance, modern pharmacological research shows that calycosin and phenylalanine could regulate glucose and lipid metabolism by influencing insulin resistance, inflammation, oxidative stress, and lipid accumulation [20,21,22]. Hesperidin and naringenin exhibit anti-inflammatory and anti-oxidation effects, regulate carbohydrate and lipid homeostasis, and contribute to the amelioration of lipid metabolism disorders and insulin resistance [23]. In addition, levistilide A primarily exerts anti-inflammatory and antioxidant effects and modulates AMPK/mTOR phosphorylation, as well as altering glucose metabolism levels [24]. Ferulic acid can also improve glucose and lipid metabolism, as well as restore pancreatic function [25]. Furthermore, the mechanism of action of coumaric acid mainly involves regulating hepatic lipid metabolism, anti-glycation, anti-oxidant effects, and modulating the expression of liver-related proteins PPAR and AMPK [26,27]. Thus, based on modern pharmacological research, QHTTF might improve glucose and lipid metabolism, relieve inflammation and oxidative stress, and alleviate insulin resistance, making it useful in the treatment of diabetes and dyslipidemia.

The animal experiment also demonstrated that QHTTF decreased the serum levels of GLU, TG, LDL-C, and FFA and significantly reduced the AUC of OGTT blood glucose in rats. This suggests that this herbal medicine could lower fasting blood glucose and abnormal lipid levels in ZDF rats. Patients with glucolipid metabolic disorders may experience increased oxidative stress and chronic inflammation due to excessive calorie consumption and insufficient physical activity. Meanwhile, as key metabolic regulators, the liver and pancreas are centrally involved in GLMDs [28]. Histopathological staining showed that model group rats exhibited aggravated pathological damage, inflammatory infiltration, and increased cell necrosis in the liver and pancreas, as well as severe lipid accumulation in hepatocytes. After QHTTF intervention, the rats showed notable improvements in islet morphology, a significant reduction in cell vacuolation and necrosis, and a decrease in hepatic lipid accumulation. These improvements in fatty degeneration were superior to those in the metformin group. Additionally, the Chinese medicine formula could reduce the liver weight and liver index in ZDF rats to some extent. Relative to the model group, QHTTF treatment exhibited effects similar to those of metformin therapy. However, body weight loss showed a notable trend in the Met group, while that of the QHTTF group was slightly reduced but not statistically significant (*p* > 0.05), indicating that the herbal medicine has limited efficacy in reducing body weight and alleviating obesity in ZDF rats. Another discovery of our present study was that QHTTF significantly reduced the serum levels of inflammatory factors IL-6, TNF-α, and the lipid peroxidation marker MDA, while increasing the levels of SOD, potentially exerting further effects on the glucose and lipid metabolism of rats. These basic pharmacological effects—including reduced GLU, TG, and LDL accumulation, alleviated FFA levels, and inhibited inflammation and oxidative stress—were consistent with previous pharmacological research on the major components of QHTTF.

In the current research, we employed network pharmacology and metabolomic analysis to investigate the potential mechanism of QHTTF against glucolipid metabolic disease. Network pharmacology is a comprehensive approach to predict the major active ingredients, targets, and related signaling pathways involving GLMDs and drugs. We identified 59 common drug-disease intersection targets and performed enrichment analysis on them. The GO enrichment analysis revealed that the relevant BP included cholesterol metabolic process, cellular response to lipopolysaccharide, response to xenobiotic stimulus, and oxidoreductase activity, among others. This is consistent with previous studies linking cholesterol metabolism and lipopolysaccharide response to insulin resistance and T2DM [29,30]. At the same time, KEGG pathway analysis provided evidence that the signaling pathways mainly involved the PPAR, AMPK pathways, insulin resistance, lipid and atherosclerosis, and alcoholic liver disease, among others. Furthermore, 11 potential core targets were acquired according to the PPI network, which mainly contained PPARα, PPARγ, TNF, ALB, ESR1, etc. Thus, our analysis suggested that the PPAR and AMPK signaling pathway and insulin resistance may play crucial roles in the therapeutic effect of QHTTF. In addition, molecular docking analysis showed that the compounds of calycosin, formononetin, phenylalanine, naringenin, hesperetin, and levistilide A have great binding affinity to PPARα and PPARγ. This indicated that the main active components of QHTTF might regulate glucolipid metabolism disorders in ZDF rats by modulating PPARα and PPARγ targets.

On the other hand, in the metabolomics results, we observed an imbalance of amino acids, fatty acids, and some gluconic acid in ZDF rats. QHTTF significantly decreased the levels of alpha-D-glucose, D-fructose, linoleic acid, D-xylose, and Pyruvaldehyde and increased the levels of D-Galactarate, Indolelactic acid, and DL-Lysine, among others. Subsequently, integrated analysis of the intersecting targets and differential metabolites revealed that they were primarily enriched in pathways including linoleic acid, fructose and mannose, galactose, fatty acid biosynthesis, and synthesis and degradation of ketone bodies. Recent studies also show that the dysregulation of linoleic acid metabolism, D-fructose, and D-glucose metabolism was associated with type 2 diabetes risk, and their excessive accumulation could lead to metabolic disturbances in T2DM [31,32]. Meanwhile, galactose and glycerolipid metabolisms were the primary disrupted pathways in diabetic conditions based on clinical trials [33]. Additionally, the heatmap revealed that most of the metabolites exhibited a positive correlation with the levels of blood glucose, lipids, inflammatory factors, and oxidative markers. Hence, by integrating network pharmacology, metabolomics, and previous pharmacological research, we hypothesized that the main ingredients of QHTTF may regulate PPAR-associated targets, AMPK, and insulin signaling proteins. This regulation could influence α-D-glucose metabolism, phosphorylcholine, linoleic acid metabolism, and D-galactarate metabolism, thereby mitigating inflammation reactions, oxidative stress, and insulin resistance to ultimately influence glucose and lipid metabolism in ZDF rats.

Consequently, to investigate the underlying mechanism of QHTTF, we performed biological experiments to confirm the results of the network analysis above. Western blot analysis suggested that QHTTF might upregulate the expression of PPARγ and PPARα, and increase the phosphorylation (p-AMPK) and total levels of AMPK. Peroxisome proliferator-activated receptors (PPARs) are important ligand-induced transcription factors that play crucial roles in glucose-lipid metabolism [34]. PPAR receptors are involved in regulating lipid and carbohydrate metabolism, homeostasis, and cellular processes such as proliferation, differentiation, and inflammation [35,36]. Within the PPAR family, three isoforms have been identified: PPARα, PPARγ, and PPARβ/δ. The role of PPARβ/δ has received less attention in research due to its limited clinical association with metabolic diseases. PPARα is mainly expressed in hepatic and skeletal muscle tissues, making a significant impact on regulating lipid catabolism [37]. Activation of PPARα promotes fatty acid oxidation and reduces blood lipid levels. Meanwhile, PPARγ is one of the primary targets for regulating glucose and lipid in the liver, and it serves as a key modulator of systemic lipid metabolism and insulin sensitivity [38]. In addition, PPARγ agonists can effectively treat diabetes and lipid metabolism disorders as insulin sensitizers. Moreover, PPARγ activation can regulate the PI3K/Akt insulin signaling pathway, which enhances insulin sensitivity, inhibits lipogenesis, and suppresses glucagon secretion, thereby improving glycolipid metabolism [39,40]. Notably, modulating the AMPK/Sirt1/PPARγ pathway has been shown to alleviate glucolipid metabolism disorders in fatty liver mice triggered by high-fat and high-fructose diets, thereby exerting hepatoprotective, anti-hyperlipidemic, and anti-inflammatory effects [41]. AMP-activated protein kinase is a central regulator of cellular energy balance and a master sensor of lipid and glucose metabolism [42]. AMPK is activated by phosphorylation at Thr172 (p-AMPK). Once activated, it can regulate multiple metabolic pathways, including promoting fatty acid oxidation and glycolysis, inhibiting fatty acid synthesis, reducing inflammation, and responding to nutrient stress to enhance insulin sensitivity [43]. Furthermore, in vivo studies confirm that sustained AMPK activation could mediate the metabolic effects of PPARβ/δ and play a crucial role in glucose and lipid metabolism, as well as improve hepatic function [44].

Another finding of our study was that QHTTF and metformin significantly increased the expression of IRS-1 and GLUT4 proteins in the pancreas. Both of them are essential proteins in the insulin signaling pathway. Insulin receptor substrate-1 (IRS-1) serves as a substrate for the insulin receptor tyrosine kinase and plays a critical role in insulin signaling. When insulin binds to its receptor, it triggers IRS-1 phosphorylation, which subsequently activates the PI3K and AKT targets, thereby promoting glucose transport into cells, regulating glucose metabolism, and maintaining normal blood glucose levels [45]. Studies have indicated that IRS-1 can coordinate skeletal muscle metabolism via the AKT and AMPK pathways. In T2DM models, the expression of p-AMPK/AMPK, IRS-1, and GLUT4 proteins also showed a similar trend, contributing to improved insulin sensitivity and glucolipid metabolism [46,47]. Additionally, in hepatic tissues, insulin can suppress gluconeogenesis and lipolysis through IRS-1, while simultaneously promoting fatty acid synthesis and the secretion of low-density lipoprotein (LDL), thereby maintaining a healthy balance of blood lipids [48]. GLUT4 is an insulin-regulated glucose transporter, primarily responsible for the uptake of glucose into adipose and muscle cells, playing an essential role in glucose homeostasis. When insulin binds to receptors on skeletal muscle and adipocyte surfaces, it activates the PI3K-AKT pathway, facilitating the translocation and fusion of GLUT4 with the plasma membrane, thereby enabling efficient glucose uptake [49]. The dysfunction or deficiency of GLUT4 can reduce cellular sensitivity to insulin, resulting in insulin resistance and subsequent disturbances in glucose metabolism [50,51]. Therefore, the inhibition of IRS-1 and GLUT4 due to various factors can result in insulin resistance and disruptions in glucose and lipid homeostasis. This study suggested that QHTTF treatment may regulate insulin resistance by upregulating IRS-1 and GLUT4 expression in pancreatic tissues, potentially indicating modulation of the insulin signaling pathway.

Nevertheless, there are also some limitations to our research that need further improvement. First, the lack of blinding during drug administration and outcome assessment could introduce potential bias. Secondly, the active ingredients of QHTTF can be identified by analyzing drug-containing serum in animal experiments, which might better reflect the biologically active components. This study was only a preliminary exploration of pharmacodynamics, and the pharmacokinetic characteristics should be explored in subsequent research. Next, we employed an untargeted approach but lacked targeted validation for key metabolites. Future studies should employ targeted mass spectrometry to confirm the absolute concentrations and changes of these metabolites, which is essential for strengthening our conclusions and enhancing the translational relevance of our findings. Moreover, other results of network and metabolic enrichment analyses could be further examined to elucidate the mechanism of QHTTF in treating glucolipid metabolism disorders. Finally, and equally important, we did not set up dosage groups for the TCM formula (QHTTF) in this study. This decision was based on the high cost and the results of preliminary experiments, which showed no clear dose-dependent effect, with the moderate dose yielding the best therapeutic outcome. However, the findings of our study are limited to the selected dosage. Further validation of the dose–response relationship via multi-dose gradient experiments is needed in future studies. At the same time, we are considering conducting the cellular experiments on QHTTF-related signaling pathways to elucidate the underlying mechanisms. This remains a considerable potential area for future investigation.

## 4. Materials and Methods

### 4.1. QHTTF Preparation

Huangqi (2304305), Baizhu (2210008), Chaihu (2304019), Zhishi (2303087), Danggui (2305003), Chuanxiong (2303011), Zhuling (2207014), and Laifuzi (2302021) were all purchased from Beijing Yanjing Pharmaceutical Co., Ltd. (Beijing, China) and used at a proportion of 5:4:4:3:3:3:2:2. All herbs were further prepared as decoctions, which were immersed in purified water for one hour, and boiled twice over medium and low heat. The herbal decoction was strained with gauze, and the filtrate was collected, then concentrated to 1 g/mL of raw drugs and refrigerated at 4 °C. The daily dosage of QHTTF administered to rats is based on the clinical daily prescription amount for adult humans: (78 g raw herbs/60 kg body weight) × 6.3 (The multiplication factor of 6.3 is a standard constant used to adjust the dosage from humans to rats). In addition, according to our preliminary experiments, the medium dose of QHTTF administered via gavage to diabetic rats was determined to be the optimal dose. Therefore, additional dose groups were not included in this study.

### 4.2. Instrumentation and Reagents

An automatic biochemical analyzer from Japan (Toshiba, TBA40FR, Kanagawa, Japan), a blood glucose meter (ACCU-CHEK Performa, Roche Diagnostics GmbH, Mannheim, Germany), Gamma radioimmunoassay counter (XH-6080, Xi’an, China), fully automatic microplate reader (MULTISKAN MK3, Thermo, Waltham, MA, USA), Benchtop High-Speed Refrigerated Centrifuge (Allegra X-30R, Beckman Coulter, Inc., Brea, CA, USA), Electric Incubator (Tianjin Taisite, DH4000A, Tianjin, China), Electrophoresis Apparatus (Servicebio BV-2, Servicebio BT-2, Wuhan, Hubei, China), Chemiluminescent Imaging and Analysis System (JUNYI MINICHEMI, Beijing, China), 3DHISTECH (Budapest, Hungary) Pannoramic (Desk/Midi/250/1000), AB Triple SCIEX TOF 6600 mass spectrometer system (SCIEX. Ltd., Shanghai, China), and Ultra-High Performance Liquid Chromatography (UHPLC) System (Thermo Scientific, Waltham, MA, USA) were used in this study.

The FBG (no. B2007), TG (no. B2001), T-CHO (no. B2002), LDL-C (no. B2004), HDL(no. B2003), and FFA (no. B1101) reagents were all acquired from Beijing Beijian New Creative Source Biotechnology Co., Ltd. (Beijing, China). Insulin (no. F01PZA) was purchased from the Beijing Northern Biotechnology Research Institute (Beijing, China). The IL6 ELISA kit (no. EMC003.96) and TNF-a ELISA (no. EMC102a.96) were obtained from NeoBioscience Technology Co., Ltd. (Beijing, China). The SOD kit (no. A001-3-2) and the MDA (no. A003-1) were purchased from Nanjing Jiancheng Bioengineering Institute (Nanjing, China). Furthermore, HE and Oil Red O staining solutions (G1016, Servicebio, Wuhan, China) were also utilized in this research.

### 4.3. The Chemical Constituent Analysis of QHTTF

The QHTTF decoction was prepared, and the sample (600 μL) was placed into a 1.5 mL Eppendorf tube. Next, we added 400 μL of pure methanol and vortexed the mixture for 10 s. Then, we took 200 μL of the aforementioned solution and added 200 μL of a 40% methanol aqueous solution and mixed for an additional 10 s. Finally, it was centrifuged for 15 min at 16,000× *g* at 4 °C, and we took the supernatant, which is the desired solution. For chromatographic analysis, a Vanquish UHPLC system with an ACQUITY HSS T3 analytical column (2.1 × 100 mm, 1.8 μm) was applied to separate the components at 35 °C. The flow rate was kept at 0.3 mL/min, and the mobile phase consisted of A (0.1% formic acid in water) with B (formic acid acetonitrile) to conduct further gradient elution. In addition, mass spectrometry data, including primary and secondary mass spectra, were analyzed by using a Q-Exactive HFX mass spectrometer integrated with the UHPLC platform. The positive and negative ionization modes were 3800 V (ESI+)/3500 V (ESI−), sheath gas flow 40 L/min, ion transport tube temperature 320 °C, and atomization temperature 350 °C, respectively.

Data analysis was conducted through XCMS software 4.6.0 for peak matching, RT adjustment, and peak area extraction. Compounds were identified by searching the local TCM high-resolution mass spectrometry database of the Institute of Biophysics, Chinese Academy of Sciences, with a primary mass error of less than 25 ppm and a secondary fragment spectrum match score greater than 0.7. Currently, it is widely accepted that a score above 0.7 ensures reliable identification.

### 4.4. QHTTF Network Pharmacology Analysis

The Herb Pharmacology database (HERB) (http://herb.ac.cn/, accessed on 6 March 2024), SwissTarget Prediction (http://www.swisstargetprediction.ch/, accessed on 6 March 2024), uniport data (https://www.uniprot.org/, accessed on 9 March 2024), and Swiss ADME (http://www.swissadme.ch/, accessed on 9 March 2024) were used to identify the targets of QHTTF, which are based on the main chemical components obtained from mass spectrometry and the Rule of Five principles (Lipinski, Ghose, Veber, Egan, and Muegge). At the same time, the selected compounds fulfilled two or more of the five criteria that were evaluated as “yes” and exhibited a high GI absorption rating. Subsequently, the potential targets of T2DM combined with hyperlipidemia or lipid metabolism disorders were searched from the Drug Bank (http://go.Drugbank.com/, accessed on 13 March 2024), OMIM (https://omim.org/, accessed on 13 March 2024), and Gene Cards (https://www.genecards.org/, accessed on 13 March 2024 ) databases. Then, all duplicates were removed, and the intersection targets along with genes of QHTTF and T2DM with hyperlipidemia were identified and visualized by a Venn diagram from the Bioinformatics web platform (https://www.bioinformatics.com.cn, accessed on 16 March 2024). Moreover, the screened overlapping targets and genes were submitted to the String database (https://string-db.org/, accessed on 16 March 2024) to build the PPI network and then visualized through Cytoscape 3.9.1 software. Finally, to verify the functions of the intersected genes, Gene Ontology and Kyoto Encyclopedia of Genes and Genomes (GO and KEGG) pathway enrichment analyses were conducted using the DAVID database (https://david.ncifcrf.gov, accessed on 18 March 2024), and the 20 most enriched pathways were represented through the Bioinformatics analysis platform.

### 4.5. Molecular Docking Analysis

The active components of QHTTF were identified through LC-MS analysis and prioritized via Lipinski’s Rule of Five and docking scores. Molecular docking against core therapeutic targets, including PPARα, PPARγ, and TNF-α, was performed using AutoDock Vina 1.5.6, with protein structures modeled via SWISS-MODEL (PDB format) and ligands prepared from public databases (SDF format). The most frequent low-energy conformations were selected and visualized using PyMOL 3.1 and Discovery Studio 2019, with the best binding and conformational robustness effect for output.

### 4.6. Animals and Drug Administration

Male and SPF-grade diabetic obese rats (Zucker Diabetic Fatty rats, ZDF) of 8 weeks of age were utilized in our study, which were purchased from Beijing Vital River Biotech Co., Ltd. (Beijing, China) and induced by high-fat diets (K5008) for 6 weeks as the research subjects. The high-fat diet K5008 (protein 26.84%, fat 16.71%, and carbohydrates 56.44%) was obtained from Beijing Keao Xieli feed Co., Ltd. (Beijing, China). The rats were housed at the experimental animal center of the Institute of Basic Theory of Traditional Chinese Medicine of the Chinese Academy of Chinese Medical Sciences under conditions of 20–26 °C, 40–70% relative humidity, with a 12 h light/dark cycle and free access to drinking water. In total, 32 ZDF rats were randomly assigned to four groups by an independent third party using a random number table generated through computer—CON (healthy control), M (diabetic model), QHTTF (traditional Chinese medicine), and MET (metformin) groups. Rats in the QHTTF group were given 10 mL/kg of Chinese herb remedy and 10 mL/kg metformin solution in the western medicine group, according to the clinical dose conversion for humans, while rats were offered the same volume of saline in the CON and Model groups. After 8 weeks of Chinese medicine treatment, the rats were sacrificed with pentobarbital. Then, the rats’ serum was collected and preserved at −80 °C, and the liver and pancreas were extracted, packaged, and rapidly fixed in 4% paraformaldehyde. Subsequently, various biochemical indicators and molecular biology tests were conducted. This experimental protocol was approved by the Ethics Committee of the Chinese Academy of Chinese Medical Sciences, approval number: IBTCMCACMS21-2303-01.

### 4.7. Metabolomics Research and Metabolite Analysis

The APT-BioCloud platform (Shanghai, China) was used for untargeted metabolomics profiling. Briefly, the frozen plasma samples were thawed at 4 °C, and then an appropriate amount of sample was mixed with a cold solution of methanol, acetonitrile, and water (in a 2:2:1 ratio), vortexed well, sonicated at low temperature for 30 min, spun in a centrifuge at 14,000× *g* at 4 °C for 15 min, and the supernatant was collected for injection and analyzing metabolites.

The serum samples in the CON, M, and QHTTF groups were chromatographically separated via an Agilent 1290 UHPLC system equipped with a HILIC column (25 °C, 0.5 mL/min flow rate, 2 μL injection volume). The mobile phase consisted of (A) 25 mM ammonium acetate/ammonia water and (B) acetonitrile, with the following gradient profile: 0–0.5 min (95% B), 0.5–7 min (65% B), 7–8 min (40% B), 8–9 min (isocratic 40% B), 9–9.1 min (95% B), and 9.1–12 min (isocratic 95% B). Meanwhile, all samples were maintained at 4 °C in the autosampler, and QC samples were inserted into the sample queue to monitor and evaluate the stability of the system and the reliability of the experimental data. An AB Triple 6600 mass spectrometer was applied for the acquisition of the first- and second-level mass spectra of the samples, after separation with Agilent 1290 UHPLC. At the same time, Electrospray Ionization (ESI) was used in both positive and negative ion modes. Moreover, the ESI source parameters were set as follows: the ion source temperature was 600 °C, and the spray voltage (ISVF) was ±5500 V for both positive and negative modes.

The data obtained from UPLC-Q-TOF-MS underwent peak detection, RT adjustment, and peak extraction via XCMS software. Then, the quality of the experimental data was evaluated and analyzed after metabolite structural identification and data preprocessing. Following peak extraction, the resulting data matrix underwent preprocessing to ensure quality and reliability. Missing values, largely resulting from ion intensities below the detection limit, were imputed using the k-nearest neighbors (KNN) algorithm. To reduce systematic variations between samples, the data were normalized by the probabilistic quotient normalization (PQN) method to balance the influence of high and low-abundance metabolites in multivariate statistical modeling. The VIP scores from OPLS-DA (threshold > 1) combined with *p*-values (<0.05) were selected to identify the differential metabolites in this research.

### 4.8. Animal Experiments

#### 4.8.1. General Biochemical Analysis

The rats’ blood samples in all groups were collected via the abdominal aorta at the 8th week and followed by centrifugation at 4 °C (3000 r, 15 min). Next, the serum levels of fasting blood glucose (FBG) and 2 h postprandial blood glucose (2 h FG), total cholesterol (TC), triglycerides (TG), free fatty acid (FFA), low-density lipoprotein cholesterol (LDL-C), and HDL-C levels were measured via an automated biochemical analyzer. The insulin bioactivity (INS) was calculated through radioimmunoassay following the manufacturer’s guidelines, while insulin sensitivity was assessed using the HOMA-IR index: [Fasting insulin (μIU/mL) × Fasting glucose]/22.5.

#### 4.8.2. Body Weight and Liver Index

The body mass and daily consumption (food and water) of the different groups of the ZDF rats were determined and recorded every two weeks, and the body weight, liver weight were analyzed at the 0th and 8th weeks. After 8 weeks’ medicine therapy, the body weight was again measured and followed by anesthesia based on their weight. Blood was collected from the abdominal aorta, the liver tissue was isolated, cleaned, and dried, and then weighed. Liver weight index = liver tissue (g)/weight (g) × 100%. Subsequently, the liver was placed in paraformaldehyde for fixation to perform pathology analysis.

#### 4.8.3. Oral Glucose Tolerance Test (OGTT)

The different groups of rats were subjected to an OGTT after a 15 h fasting period and administered a 50% glucose solution by gavage (2 g/kg body weight). The rats’ serum was taken from the tail vein later at 0, 30, 60, 90, and 120 min by using an ACCU-CHEK Performa glucose meter to monitor the glucose levels.

#### 4.8.4. Histopathological Observation and Analysis

After blood collection via abdominal aorta puncture, pancreas and liver tissue were isolated from different groups of rats and fixed in 4% paraformaldehyde. Then washed via saline solution and immersed in 75% ethanol overnight, and afterward, tissue trimming, dehydration, embedding with paraffin, sectioning, and H&E staining were carried out, followed by mounting for microscopic observation. In addition, frozen sections of the rat liver and pancreas tissues were performed using an optical coherence tomography (OCT) compound to embed, with a section thickness of 8 µm, then incubated with Oil Red O staining solution and counterstained with hematoxylin to evaluate for any pathological alterations.

#### 4.8.5. Inflammatory Cytokine and Antioxidant Capability Detection

The blood samples that were collected from different ZDF rat groups were centrifuged and separated at 3000 rpm for 15 min. The obtained sera were used to assess the levels of inflammatory factors and oxidative stress. The concentrations of TNF-α and IL-6 factors were determined using ELISA kits from NeoBioscience Technology Co., Ltd (Shenzhen, China). The SOD activity and MDA contents in serum were quantified with detection kits from Nanjing Jiancheng Institute to evaluate the antioxidant capability of drugs. All the experiments were executed following the manufacturer’s guidelines.

#### 4.8.6. Immunohistochemical Detection of Insulin Pathway-Related Proteins

The pancreas tissue isolated from different rat groups was preserved in 4% paraformaldehyde, followed by embedding in paraffin, and sectioned with 5 μm for an immunohistochemical (IHC) staining experiment. For each rat, three non-consecutive sections from the pancreas were analyzed, and four random, non-overlapping fields of view (20× magnification) from each section were captured and quantified. The sections were incubated with the IRS-1 (Servicebio, GB111506, Wuhan, Hubei, China) and GLUT4 (Immunoway, YT5523, Beijing, China) antibodies at 4 °C overnight after dewaxing, antigen retrieval, endogenous enzyme inhibition, and serum blocking procedure. Subsequently, the sections were exposed to horseradish peroxidase (HRP)-linked secondary antibodies for 1 h at 37 °C, then stained with a DAB kit and counterstained with hematoxylin, dehydrated, and mounted. Ultimately, the slices were viewed and scanned under Pannoramic MIDI microscope and analyzed with Image-Pro Plus 6.0 software (Media Cybernetics, Inc., Rockville Pike, MA, USA).

#### 4.8.7. Western Blot Analysis

The homogenates of hepatic tissues (50 mg) from different ZDF rat groups were dissolved on ice in RIPA lysate (1:10) and protease inhibitors to extract relevant proteins. Then, the total protein extract was centrifuged for 10 min (12,000 rpm, 4 °C) to obtain the supernatant, and the protein concentration was quantified with a BCA assay kit (Solarbio, Beijing, China). The samples (within total protein-30 μg) were separated by SDS-PAGE and electroblotted onto PVDF membranes (Millipore, Bedford, MA, USA) for 40 min, which were incubated later in TBST containing skim milk (Servicebio Technology, Wuhan, China) for 1 h at 37 °C. Subsequently, the PPARα (Immunoway, YT3835, Beijing, China), PPARγ (Proteintech, 16643-1-AP, Wuhan, China), AMPK (Immunoway, YT0216, Beijing, China), and p-AMPK (Immunoway, YP0575) antibodies were added (diluted 1:1000) and reacted at 4 °C all night. The β-actin (ABclonal, AC026, Wuhan, China) was diluted 1:100,000 and incubated overnight at 4 °C. Following that, the protein band intensities were quantified using Image J software (https://imagej.net/ij/) and normalized to the β-actin loading control. The next day, the secondary antibody was diluted in TBST (1:10,000) and incorporated into the protein bands, incubated for 60 min at room temperature, and then ECL luminescent liquid was added to expose and image through Chemiluminescent Gel Imaging and Analysis System (JY-Clear ECL, Beijing Junyi Oriental Co., Ltd., Beijing, China).

### 4.9. Statistical Analysis

The results were expressed as the mean ± standard deviation ($\bar{x}\pm s$) and analyzed using SPSS 26.0 software. One-way analysis of variance (ANOVA) was performed to determine statistical significance in comparison with the respective control for each experiment. GraphPad Prism 9.5.1 software was utilized to visualize the statistical data, and *p*-value < 0.05 was regarded as statistically significant.

## 5. Conclusions

In summary, QHTTF attenuates glucolipid metabolic disorders in ZDF rats by lowering fasting blood glucose and abnormal lipid levels, suppressing inflammation, alleviating oxidative stress, thereby improving insulin resistance and relieving liver and pancreatic tissue damage. Mechanistically, integrated network pharmacology and in vivo experimental validation suggest that QHTTF may exert its effects by upregulating hepatic PPAR and AMPK signaling pathways, as well as key insulin signaling proteins, including IRS-1 and GLUT4. Additionally, combining with metabolomics, QHTTF could regulate α-D-glucose, linoleic acid, phosphorylcholine metabolites, and metabolic pathways, including fructose and mannose metabolism, linoleic acid metabolism, and galactose metabolism. These effects are likely mediated through the modulation of PPARα and PPARγ expression. Therefore, our findings on the underlying mechanism of QHTTF provide preliminary experimental basis for clinical application of Chinese medicine formulas and support its potential as a modern TCM agent for treating T2DM with dyslipidemia as a potential agent.

## Figures and Tables

**Table 1:** The characteristics of the 22 main chemical components in QHTTF.

No	Compound	RT/min	*m*/*z*	ppm	Adduct	SuperClass
2	Phenylalanine	2.39	166.0863	18.4	[M+H]^+^	Small peptides
4	Butein	3.45	163.0390	0.98	[M+H-C_6_H_6_O_2_]^+^	Flavonoids
5	Sinapine cation	3.74	310.1648	0.3	[Cat]^+^	Phenylpropanoids (C6-C3)
6	Ferulic acid	5.00	195.0654	1.3	[M+H]^+^	Phenylpropanoids (C6-C3)
8	Hesperetin	5.29	303.0864	0.9	[M+H]^+^	Flavonoids
11	Calycosin	7.10	285.0751	1.1	[M+H]^+^	Isoflavonoids
13	9-Octadecenoic acid, 5,8,11- trihydroxy-	8.81	295.2269	0.2	[M+H-2H_2_O]^+^	Octadecanoids
14	Formononetin	9.52	269.0810	1.7	[M+H]^+^	Isoflavonoids
16	Sinensetin	10.04	373.1283	0.4	[M+H]^+^	Flavonoids
17	Nobiletin	10.79	403.1387	0.0	[M+H]^+^	Flavonoids
18	5-Methoxysalvigenin	10.83	343.1177	0.4	[M+H]^+^	Flavonoids
20	Levistilide A	14.08	381.2062	0.1	[M+H]^+^	Cyclic polyketides
25	4-Formyl-2-hydroxybenzoic acid	3.87	165.0186	4.8	[M-H]^−^	Phenolic acids (C6-C1)
26	Homogentisic acid	3.94	167.0343	4.3	[M-H]^−^	Phenolic acids (C6-C1)
27	Coumaric acid	4.69	163.0393	4.9	[M-H]^−^	Phenylpropanoids (C6-C3)
30	Azelaic acid	5.71	187.0970	2.7	[M-H]^−^	Fatty Acids and Conjugates
31	Menthyl salicylate	6.23	137.0235	6.4	[M-H-C_10_H_18_]^−^	Monoterpenoids
33	7-Hydroxy-3′-methoxyflavone	6.95	267.0664	0.5	[M-H]^−^	Flavonoids
34	1-(3,4-Dihydroxy-2,6-dimethoxyphenyl)-3-phenylprop-2- en-1-one	7.30	299.0927	0.0	[M-H]^−^	Flavonoids
35	Naringenin	8.11	271.0613	0.7	[M-H]^−^	Flavonoids
36	FA 18:1+3o	8.82	329.2335	6.9	[M-H]^−^	Octadecanoids
39	(Z)-6,9,10-Trihydroxyoctadec-7-enoic acid	11.93	311.2231	0.3	[M-H-H_2_O]^−^	Octadecanoids

No: Serial number; Compound: Name of the compound; RT (min): Retention time/min; *m*/*z*: Mass-to-charge ratio of the parent ion; ppm: Primary mass deviation; Adduct: Ionization adduct form (e.g., [M+H]^+^, [M-H]^−^); SuperClass: Compound classification.

## Data Availability

All data presented in our study can be found in this article and the Supplementary Materials.

## Supplementary Materials

The following supporting information can be downloaded at: https://www.mdpi.com/article/10.3390/ph18091347/s1, Table S1 The characteristics of total 39 main chemical constituents in QHTTF. Table S2 The characteristics of different metabolites of QHTTF treatment in ZDF rats. Figure S1 The Pos and Neg Ion Mode OPLS-DA Permutation Test Plot in metabolomics.
