# Supplementary material for: Effect and Mechanism of Qihua Tongtiao Formula (QHTTF) on Improving Glucose and Lipid Metabolism Disorders in ZDF Rats by Integrating Network Pharmacology, Metabolomics, and Biological Validation
Source: Pharmaceuticals (Basel). 2025 Sep 8;18(9):1347. doi: 10.3390/ph18091347 (PMC12472807; doi:10.3390/ph18091347)
Supplement: Supplementary file 1 [file pharmaceuticals-18-01347-s001.zip › pharmaceuticals-3816318-supplementary.pdf]

Supplementary Materials

Table S1 The characteristics of total 39 main chemical constituents in QHTTF.
Table S2 The characteristics of different metabolites of QHTTF treatment in ZDF rats.
Figure S1 The Pos and Neg Ion Mode OPLS-DA Permutation Test Plot in metabolomics.

Table S1. The characteristics of total 39 main chemical constituents in QHTTF

No	Compound_EN	RT/min	m/z	ppm	score	Adduct	SuperClass
1	Methyl 4-fluoro-1H-pyrrole-2-carboxylate	2.26	144.0477	16.3	0.8085	[M+H]+	Proline alkaloids
2	Phenylalanine	2.39	166.0863	18.4	0.9998	[M+H]+	Small peptides
3	3-Indolyllactic acid	3.06	188.0705	0.2	0.9799	[M+H-H2O]+	Tryptophan alkaloids
4	Butein	3.45	163.0390	0.98	0.9898	[M+H-C6H6O2]+	Flavonoids
5	Sinapine cation	3.74	310.1648	0.3	0.9855	[Cat]+	Phenylpropanoids (C6-C3)
6	Ferulic acid	5.00	195.0654	1.3	0.9972	[M+H]+	Phenylpropanoids (C6-C3)
7	Sibiricose A6	5.19	369.1182	0.3	0.9763	[M+H-C6H12O6]+	NA
8	Hesperetin	5.29	303.0864	0.9	0.9931	[M+H]+	Flavonoids
9	6,7-Dihydroxyligustilide	6.18	207.1015	0.6	0.9824	[M+H-H2O]+	Cyclic polyketides
10	3-(4-Methoxyphenyl)-4-oxo-4Hchromen-7-yl 6-O-(carboxyacetyl)-.beta.-Dglucopyranoside	6.94	517.1344	0.7	0.9923	[M+H]+	Isoflavonoids
11	Calycosin	7.10	285.0751	1.1	0.9962	[M+H]+	Isoflavonoids
12	PAz-PC	8.62	704.3979	11.6	0.7287	[M+K]+	Glycerophospholipids
13	9-Octadecenoic acid, 5,8,11-trihydroxy-	8.81	295.2269	0.2	0.9065	[M+H-2H2O]+	Octadecanoids
14	Formononetin	9.52	269.0810	1.7	0.9996	[M+H]+	Isoflavonoids
15	7,8-Dimethoxycoumarin	9.86	207.0654	0.7	0.9968	[M+H]+	Coumarins
16	Sinensetin	10.04	373.1283	0.4	0.9786	[M+H]+	Flavonoids
17	Nobiletin	10.79	403.1387	0.0	0.9963	[M+H]+	Flavonoids
18	5-Methoxysalvigenin	10.83	343.1177	0.4	0.9763	[M+H]+	Flavonoids
19	Ligustilide A	12.31	191.1067	0.9	0.9983	[M+H]+	Cyclic polyketides
20	Levistilide a	14.08	381.2062	0.1	0.9986	[M+H]+	Cyclic polyketides
21	N-Carbamoyl-L-phenylalanine	2.40	164.0710	4.2	0.9955	[M-H-CHNO]-	Small peptides
22	Caffeoyl quinic acid	2.95	353.0882	3.5	0.9812	[M-H]-	Phenylpropanoids (C6-C3)
23	1,3,4-Trihydroxy-5-(((2E)-3-(2-(4-hydroxy-3-methoxyphenyl)-3-(hydroxymethyl)-7-methoxy-2,3-dihydro-1-benzofuran-5-yl)prop-2-enoyl)oxy)cyclohexanecarboxylic acid	3.46	191.0556	2.8	0.9988	[M-H-C20H18O6]-	Lignans

24	Vicenin-2	3.74	593.1518	1.3	0.9905	[M-H]-	Flavonoids
25	4-Formyl-2-hydroxybenzoic acid	3.87	165.0186	4.8	0.9989	[M-H]-	Phenolic acids (C6-C1)
26	Homogentisic acid	3.94	167.0343	4.3	0.9991	[M-H]-	Phenolic acids (C6-C1)
27	Coumaric acid	4.69	163.0393	4.9	0.9992	[M-H]-	Phenylpropanoids (C6-C3)
28	Narirutin	4.96	579.1722	0.1	0.9985	[M-H]-	Flavonoids
29	3',6-Disinapoylsucrose	5.21	753.2253	1.7	0.9982	[M-H]-	Phenylpropanoids (C6-C3)
30	Azelaic acid	5.71	187.0970	2.7	0.9972	[M-H]-	Fatty Acids and Conjugates
31	Menthyl salicylate	6.23	137.0235	6.4	0.9989	[M-H-C10H18]-	Monoterpenoids
32	Didymin	6.74	593.1881	1.4	0.9956	[M-H]-	Flavonoids
33	7-Hydroxy-3'-methoxyflavone	6.95	267.0664	0.5	0.9933	[M-H]-	Flavonoids
34	1-(3,4-Dihydroxy-2,6-dimethoxyphenyl)-3-phenylprop-2-en-1-one	7.30	299.0927	0.0	0.9223	[M-H]-	Flavonoids
35	Naringenin	8.11	271.0613	0.7	0.9967	[M-H]-	Flavonoids
36	FA 18:1+3o	8.82	329.2335	6.9	0.9934	[M-H]-	Octadecanoids
37	4-Hydroxy-3-(3-methylbut-2-enyl)benzoic acid	10.03	205.0866	2.1	0.9241	[M-H]-	Phenolic acids (C6-C1)
38	Saikosaponin B2	10.24	825.4648	0.5	0.8948	[M+FA-H]-	Triterpenoids
39	(Z)-6,9,10-Trihydroxyoctadec-7-enoic acid	11.93	311.2231	0.3	0.9518	[M-H-H2O]-	Octadecanoids

No: Serial number; **Compound:** name of the compound; **RT (min):** Retention time/min; **m/z:** Mass-to-charge ratio of the parent ion; **ppm:** Primary mass deviation; **Score:** Spectral Match Score; **Adduct:** Ionization adduct form (e.g., $[M+H]^+$, $[M-H]^-$); **SuperClass**: Compound classification

Table S2. The characteristics of different metabolites of QHTTF treatment in ZDF rats

No	Metabolites	RT/s	HMDB ID	Fold Change	P value	VIP
1	1-Methylnicotinamide	310.5350	H0000699	0.58	0.0014	3.38
2	1-myristoyl-sn-glycero-3-phosphocholine	197.3430	H0010379	1.45	0.0116	3.70
3	Arg-Gln-Arg	440.5460	NA	0.42	0.0315	1.81
4	D-Mannose	289.9540	H0000169	0.76	0.0438	2.84
5	D-ornithine	305.4960	H0003374	1.28	0.0139	5.59
6	DL-Lysine	530.1400	H0142894	1.35	0.0054	2.63
7	Dl-normetanephrine	31.9155	H0000819	1.48	0.0481	1.93
8	Ectoine	330.4335	NA	0.62	0.0083	1.41
9	Ile-Tyr	306.1535	NA	0.50	0.0069	1.81
10	L-Serine	335.0120	H0000187	0.65	0.0060	2.64
11	L-thiocitrulline	384.5525	NA	0.52	0.0061	1.54
12	L-Tryptophan	247.8590	H0000929	0.69	0.0099	1.51
13	Meperidine	523.0300	H0014597	0.74	0.0313	7.85
14	Val-Ala-Lys	261.1075	NA	0.66	0.0193	3.12
15	.gamma.-linolenic acid	43.0895	H0003073	0.85	0.0467	2.91
16	2-keto-D-Gluconic acid	212.1115	NA	0.49	0.0064	4.29
17	2-ketohexanoic acid	304.9450	H0001864	0.44	0.0233	1.46

18	7.alpha.,17.alpha.-dimethyl-5.beta.-androstane-3.alpha.,17.beta.-diol glucuronide	29.2060	NA	0.59	0.0117	3.46
19	Acetoacetic acid	296.3360	H0000060	0.74	0.0183	2.40
20	Alpha-D-Glucose	297.4995	H0003345	0.73	0.0022	7.90
21	alpha,alpha-Trehalose	361.5980	NA	0.15	0.0317	1.88
22	Blood group b trisaccharide	375.8830	NA	0.60	0.0114	1.63
23	Cholesteryl sulfate	29.0590	H0000653	0.55	0.0086	15.68
24	D-Aspartic acid	299.7645	H0006483	2.16	0.0346	1.38
25	D-fructose	295.9660	H0062538	0.63	0.0038	28.13
26	D-Galactarate	109.2000	H0000639	3.05	0.0459	2.10
27	D-lyxose	109.9890	NA	0.81	0.0094	3.30
28	D-Tagatose	321.5600	H0003418	0.66	0.0198	1.10
29	D-xylose	296.2180	H0060254	0.67	0.0262	3.39
30	Dronedarone	116.0210	NA	0.59	0.0320	1.29
31	Farrerol	296.4720	H0130571	0.73	0.0313	1.78
32	Glycerophosphate(2)	383.8705	H0000126	0.61	0.0198	2.48
33	Indolelactic acid	118.7160	H0000671	1.19	0.0260	2.74
34	L-threonate	305.6490	H0000943	0.66	0.0084	1.16
35	Linoleic acid	42.9935	H0000673	0.78	0.0025	17.11
36	Mitragynine	30.3970	H0041933	0.71	0.0262	1.73
37	Nervonic acid	42.1340	H0002368	1.39	0.0387	1.15
38	Phenaceturic acid	190.3020	H0000821	2.16	0.0100	2.37
39	Phosphorylcholine	383.7560	H0001565	0.57	0.0284	4.40
40	Pristimerin	29.4315	NA	0.67	0.0152	4.34
41	Pyrrolidinium, 1-[(7r)-7-(acetyloxy)-4-hydroxy-4-oxido-3,5,9-trioxa-4-phosphapentacos-1-yl]-1-methyl-,inner salt	176.8840	NA	0.63	0.0336	1.31
42	Pyruvaldehyde	296.9400	H0001167	0.73	0.0157	3.89
43	Rauwolscine	29.7765	NA	0.76	0.0459	2.13
44	Taurine	291.7200	H0000251	0.86	0.0061	10.01

Figure S1

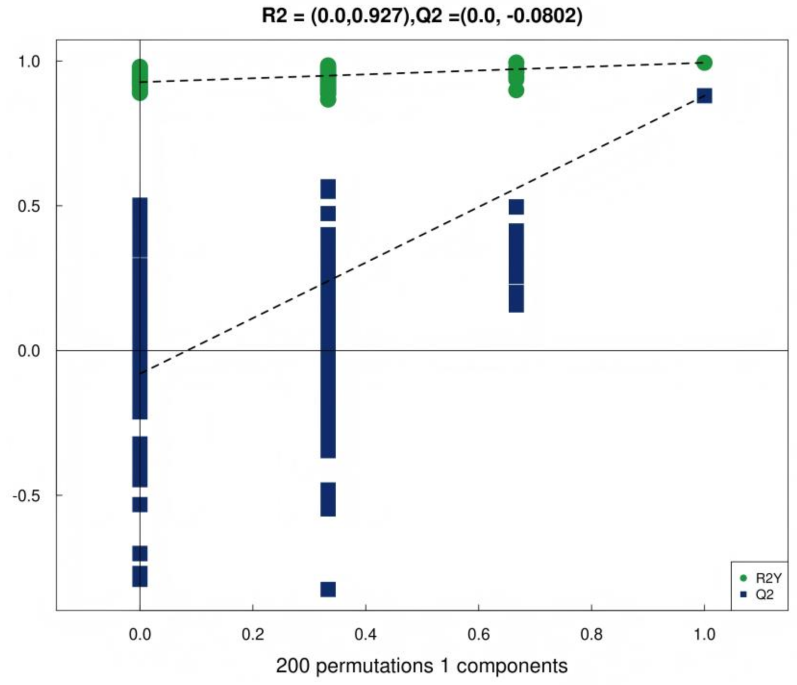

A: model vs normal

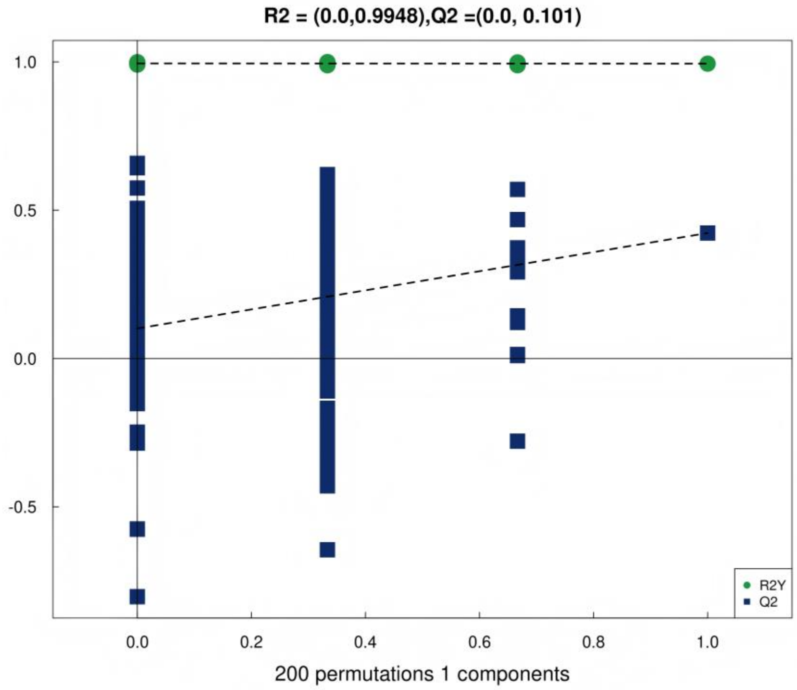

B: QHTTF vs model

Figure S1. The OPLS-DA Permutation Test Plot in positive modes (A-B).

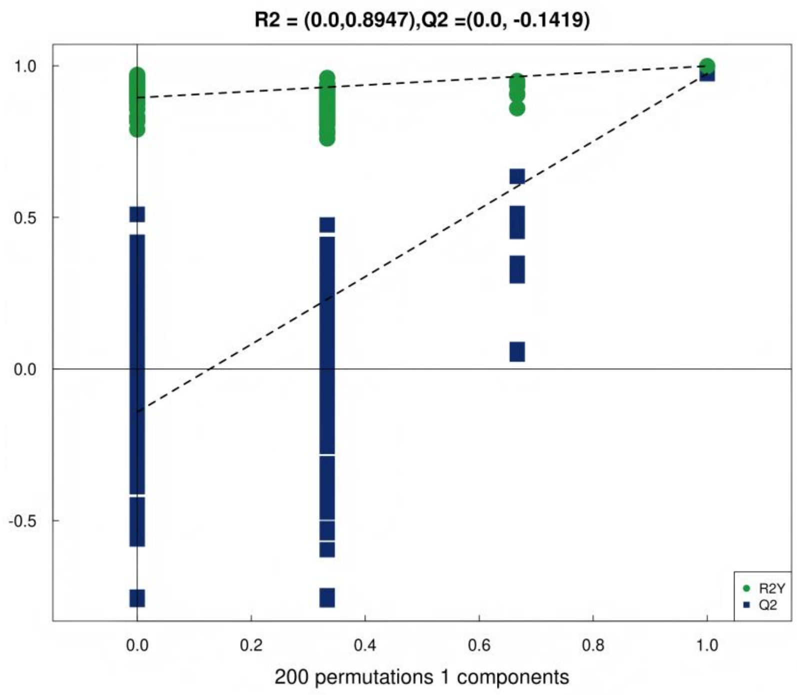

C: model vs normal

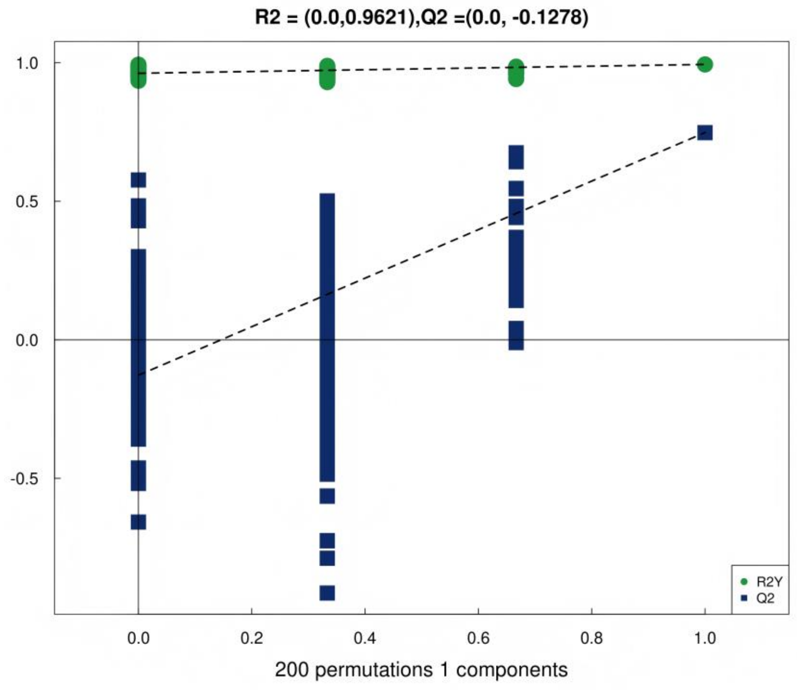

D: QHTTF vs model

Figure S1. The OPLS-DA Permutation Test Plot in negative modes (C-D).
